# Prostate-specific membrane antigen expression in hepatocellular carcinoma: potential use for prognosis and diagnostic imaging

**DOI:** 10.18632/oncotarget.27024

**Published:** 2019-06-25

**Authors:** Yuri Tolkach, Diane Goltz, Anika Kremer, Hojjat Ahmadzadehfar, Dominik Bergheim, Markus Essler, Marnix Lam, Bart de Keizer, Hans-Peter Fischer, Glen Kristiansen

**Affiliations:** ^1^ Institute of Pathology, University Hospital Bonn, Bonn 53127, Germany; ^2^ Clinic of Nuclear Medicine, University Hospital Bonn, Bonn 53127, Germany; ^3^ Department of Radiology and Nuclear Medicine, University Medical Center Utrecht, Utrecht 3584 CX, the Netherlands

**Keywords:** *FOLH1*, hepatocellular carcinoma, PET/CT, prostate-specific membrane antigen, theranostic

## Abstract

**Aim:**

The prostate-specific membrane antigen (PSMA) is currently being established as a potent diagnostic marker in many tumor types. So far, its evidence in hepatocellular carcinoma (HCC) is sparse. The aim of our study was a comprehensive evaluation of PSMA expression and its prognostic role in patients with hepatocellular carcinoma as well as feasibility test of PSMA as an agent for diagnostic imaging.

**Methods:**

The cohort for immunohistochemistry consisted of 153 patients with HCC. For validation purposes the HCC cohort (n = 359) of The Cancer Genome Atlas was analyzed on transcript level as well.

**Results:**

On immunohistochemistry, non-tumorous liver tissue showed PSMA expression on canalicular membranes in all cases. In tumor tissue two patterns of expression, with a canalicular (41.1% of tumors) and a neovascular (89.9% of tumors) staining were seen. Completely negative for both two patterns were only 4.1% of tumors; conversely, 79.2% of the tumors showed high levels of PSMA protein expression at any location. At mRNA level higher *FOLH1* (PSMA) expression rates were statistically significant and independently associated with longer overall survival times.

Additionally, a case report of successful diagnostic ^68^Ga-PSMA-11 PET/CT in a patient with HCC progression on multiple therapy lines is provided.

**Conclusions:**

Majority of hepatocellular carcinomas show high levels of PSMA expression on tumor vessels and on canalicular membrane of the tumor cells. Putative diagnostic, prognostic and therapeutic value of PSMA in HCC warrants further clinically oriented investigations.

## INTRODUCTION

The prostate-specific membrane antigen (PSMA) is currently being established as a potent diagnostic marker in many tumor types [1]. Many normal tissue types (kidney, liver, salivary and lacrimal glands, breast and prostate tissue) and tumor entities other than prostate cancer express PSMA, mostly on endothelial cells of tumor vessels [2–6].

**Figure 1:**
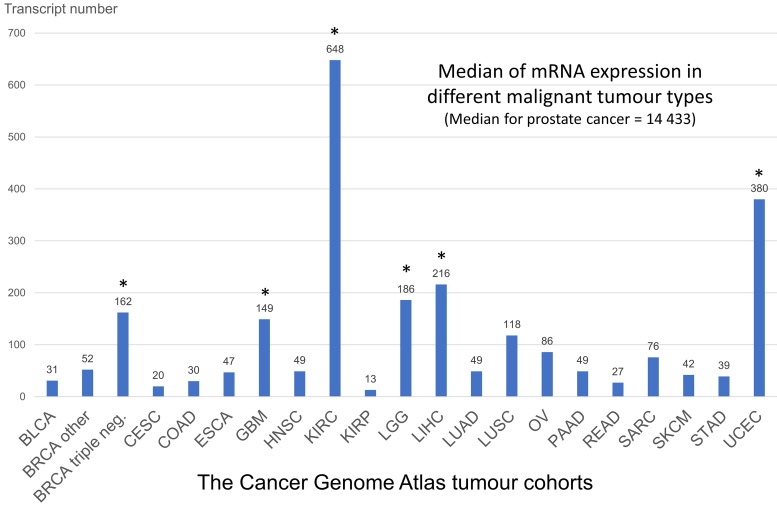
Summary analysis of FOLH1 (PSMA) mRNA expression in different tumor types of The Cancer Genome Atlas (see methods). The outlier tumors with higher than average FOLH1 mRNA expression are shown with asterix (^*^). These tumors are potential targets for PSMA based diagnostics and treatment. Abbreviations: BLCA – bladder cancer, BRCA – breast cancer (dichotomized for groups with triple-negative breast cancer and all other cases), CESC – cervix squamous cell carcinoma, COAD – colon adenocarcinoma, ESCA – esophageal carcinoma, GBM – glioblastoma, HNSC – head and neck squamous carcinoma, KIRC – clear-cell renal cell carcinoma (RCC), KIRP – papillary RCC, LGG – low-grade glioma, LIHC – hepatocellular carcinoma, LUAD – lung adenocarcinoma, LUSC – lung squamous cell carcinoma, OV – ovarian carcinoma, PAAD – pancreas adenocarcinoma, READ – rectum adenocarcinoma, SARC – sarcomas, SKCM – skin cutaneous melanoma, STAD – stomach adenocarcinoma, UCEC – uterine corpus endometrial carcinoma.

At the mRNA level PSMA-coding gene *FOLH1* (folate hydrolase 1) expression is to a certain extent detectable almost in all tumor types, however, there are several spikes on this pan-tumor landscape where the potential diagnostic and therapeutic applications of PSMA seems to be more promising, even if the expression in comparison to prostate cancer is relatively low (Figure 1). Some of these tumors have already been extensively characterized with regard to PSMA expression (kidney cancer, breast cancer, gastric cancer, colon cancer), others are still not addressed (hepatocellular carcinoma, endometrial cancer). Several case reports and one case series to date reported the positivity at PSMA positron emission tomography / computed tomography (PET / CT) in approximately 10 patients with a hepatocellular carcinoma (HCC) [7–10].

This study provides the first comprehensive evaluation of the PSMA expression at protein and mRNA levels in benign and malignant liver tissue of patients with hepatocellular carcinoma, as well as in patients with benign liver tumors. It also demonstrates a prognostic role of PSMA in patients with HCC. This is further complemented by a case report of successful diagnostic ^68^Ga-PSMA-11 PET/CT application in a patient with HCC after multiple therapy lines.

## RESULTS

### PSMA expression patterns in benign and tumor tissue (Immunohistochemistry cohort)

In general, high levels of expression could be seen in both benign peritumoral and tumor tissue.

**Figure 2:**
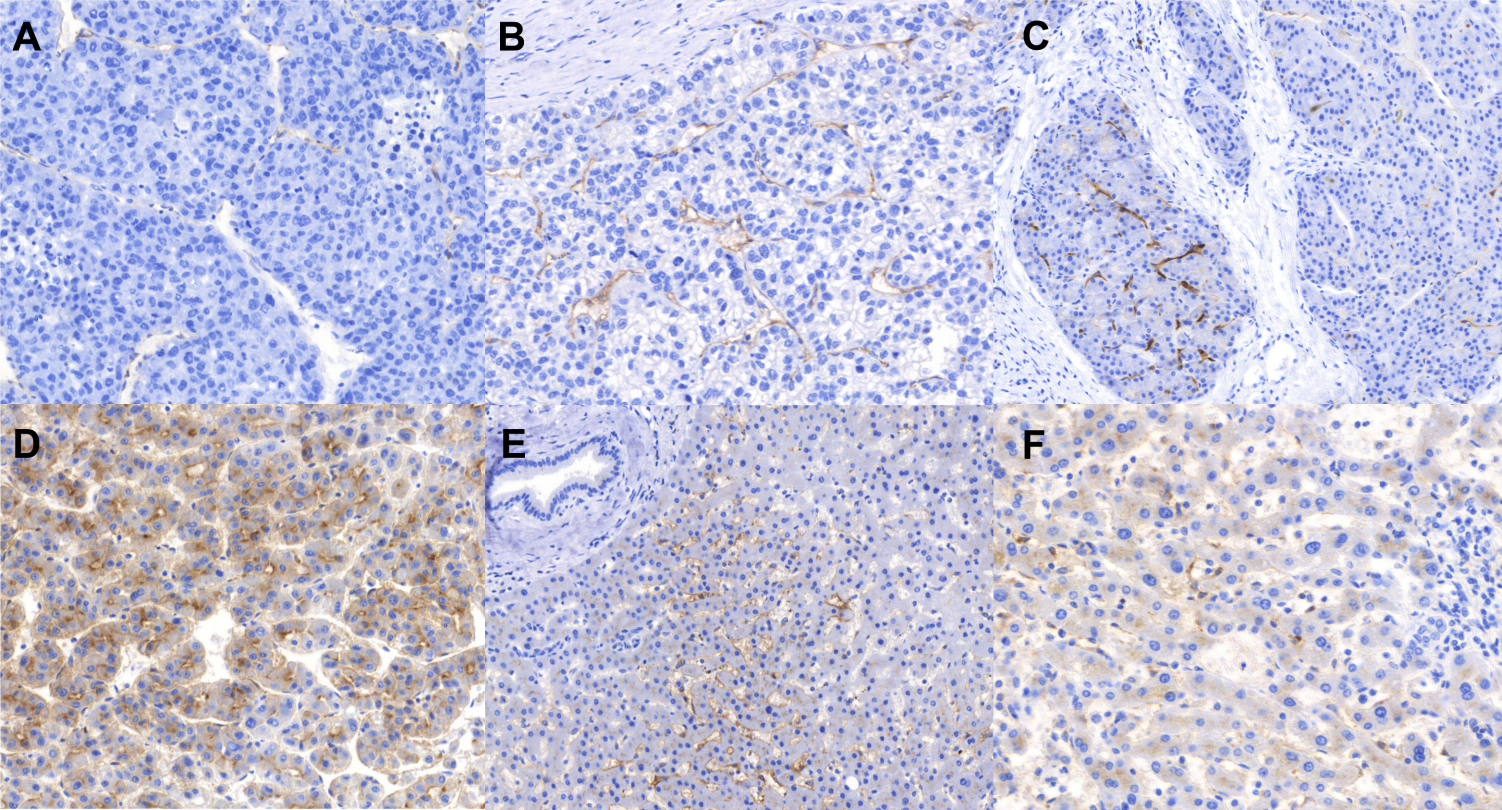
Immunohistochemistry staining patterns of PSMA. **(A)** Tumor tissue: Negative and weak neovascular expression (staining intensities “0” and “1”), **(B)** Tumor tissue: Moderate neovascular staining intensity (“2”), **(C)** Tumor tissue: Neovascular strong staining intensity on the left side (“3”) and weak expression (“1”) on the right side illustrating intratumoral heterogeneity, a relatively frequent event, **(D)** Tumor tissue: a very strong (“3”) canalicular pattern, **(E, F)** Benign peritumoral tissue showing canalicular pattern (E, F) as well as staining of single intrasinusoidal Kupffer cells.

In non-tumoral tissue (available corresponding to 129 tumors) only canalicular pattern and occasionally staining of intrasinusoidal macrophages (Kupffer cells) was evident (Figure 2). All cases were positive with 67.4% showing moderate and high levels of expression (intensity “2” and “3”); on average 80% of canalicular structures were positive. Similar levels of expression were present in cirrhotic vs non-cirrhotic non-tumoral liver tissue (*p*>0.05).

**Figure 3:**
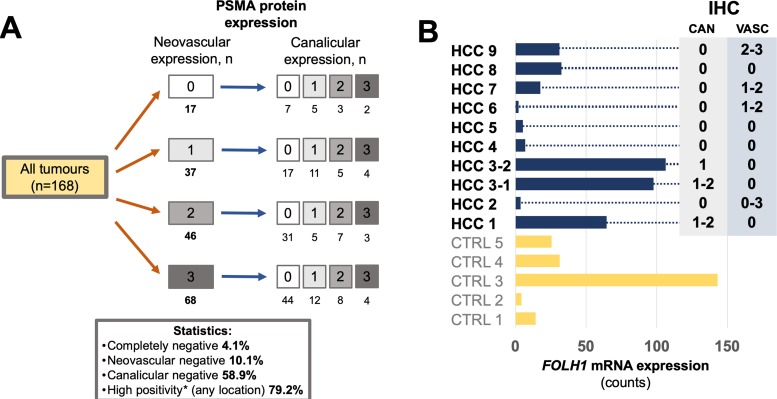
**(A)** PSMA protein expression in 168 tumors from 153 patients with hepatocellular carcinoma HCC (immunohistochemistry cohort), illustrating stratification according to two different staining patterns of tumor tissue (neovascular and canalicular). In general, HCCs demonstrate very high levels of PSMA positivity. **(B)** Comparison of PSMA protein expression and FOLH1 mRNA expression (absolute expression, measured in counts) in 10 representative HCC from 9 patients (immunohistochemistry cohort) and 5 non-matched benign liver samples (CTRL). Whole block sections were used for analysis. For immunohistochemical protein expression (right side) numbers represent staining intensity (semiquantitative: “0” – no expression, “1” – weak, “2” – moderate, “3” – strong expression), ranges outline intratumoral heterogeneity of corresponding staining pattern. Abbreviations: IHC – immunohistochemistry, CAN – canalicular staining pattern, VASC – neovascular staining pattern.

Tumor tissue from patients with HCC showed two distinct staining patterns (Figure 2, Figure 3): neovascular and canalicular (correlation between both: Pearson r=-0.15, p=0.052). The latter was mostly evident in tumors capable of building of acinar / tubular structures and present in 41.1% of tumors. High levels of neovascular positivity were present (89.9% of tumors). Only 4.1% of tumors were completely negative for both two patterns.

The percentage of positive vessels and intensity of their staining were highly correlated; similarly, percentage of positive tumor cells (canalicular pattern) was highly correlated to the intensity canalicular staining (for both patterns Pearson r > 0.89, p < 2.2e-16). A weak positive correlation was present between maximal PSMA expression in tumor tissue (neovascular pattern only) and in the corresponding non-tumoral tissue (Pearson r = 0.19, p = 0.013).

PSMA expression showed some intratumoral heterogeneity in cases with positive expression. High levels of heterogeneity (differences between tumor spots > 1 expression intensity tier) for canalicular pattern were evident in 13.0% of tumors, low levels (difference = 1 expression intensity tier) in 34.8% of tumors. For neovascular expression high levels of intratumoral heterogeneity were present in 13.9% of tumors, low levels in 45.0% of tumors.

PSMA protein expression was also analyzed in 13 focal nodular hyperplasia (FNH) cases and in 11 inflammatory hepatic adenomas (IHA). Analogous to benign liver tissue with rather weak staining (intensity “1”), only the canalicular pattern was evident in all FNH and IHA cases with three cases of IHA and seven cases of FNH showing focal stronger expression at canalicular membranes (intensity “2”).

Comparisons between protein (immunohistochemistry) and mRNA expression were performed on whole block sections of tumor (n=10) and benign (n=5) tissue (Figure 3B). In most positive HCC cases PSMA protein expression showed heterogeneity for both neovascular and canalicular patterns and was correlated to mRNA expression. There was a trend to increased overall mRNA expression in tumors showing canalicular pattern, also compared to mRNA expression in benign tissue.

### Correlation of expression with clinicopathological parameters (Immunohistochemistry cohort)

No associations were revealed for any of two tumoral PSMA expression patterns with clinicopathological parameters such as age, gender, pT-stage of the tumor and tumor size, presence of vascular invasion, metastatic disease, etiology and presence of liver cirrhosis.

The only exception was lower canalicular PSMA expression in patients with higher histological grade of the tumor (Fisher’s exact test p = 0.018): 27.9% of Grades 1/2 tumors had high levels of expression (intensity “2” and “3”) compared to 10.9% of tumors with Grades 3/4. No association was found between neovascular PSMA expression and histological grade of the tumor.

### Correlation of mRNA expression with clinicopathological parameters (The Cancer Genome Atlas cohort)

*FOLH1* mRNA expression and clinical information were available for 359 patients with HCC in The Cancer Genome Atlas (TCGA) cohort (Supplementary Table 1). *FOLH1* mRNA expression was higher in benign liver tissue (measured in normalized reads: mean 1034, range 206-1932) than in tumor (mean 432, range 4-3585) tissue (t-test p<0.0001). Only 10.3% of the tumor samples demonstrated FOLH1 mRNA expression more than average of that in normal tissue. The only statistically significant correlation for *FOLH1* mRNA expression in tumor tissue was with histological grade of the tumor (Pearson’ s r = -0.13, p = 0.011).

### Survival analysis (Immunohistochemistry and TCGA cohort)

No associations were found in our relatively small immunohistochemistry cohort (n=107, number of events = 26) between PSMA protein expression (both patterns) and overall survival of patients with HCC, even with pooling of patients from several staining intensity groups.

**Figure 4:**
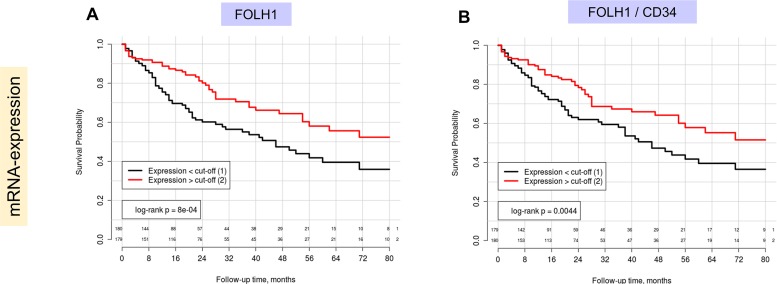
Kaplan-Meier estimates with corresponding log-rank test p-values for FOLH1 (PSMA) mRNA expression in patients of TCGA cohort (Overall survival as endpoint, number of patients = 359, number of events = 125): **(A)** Raw FOLH1 mRNA expression, **(B)** FOLH1 mRNA expression normalized through mRNA expression of vascular marker (CD34). In both A) and B) median expression levels serves as cut-off for patient dichotomization. Lower PSMA expression is associated with negative outcome.

At the mRNA level *FOLH1* (PSMA) expression was highly statistically significant associated with the overall survival in Kaplan-Meier, univariate and multivariate (pT-stage, ECOG (Eastern Cooperative Oncology Group) performance status, presence of cirrhosis, postoperative ablation therapy) Cox regression analysis, using median of expression for dichotomization (Figure 4, Table 2). Both raw *FOLH1* expression (Table 2) and vascular *FOLH1* expression (Supplementary Table 3) normalized through CD34 were statistically significant in multivariate models. At that, lower mRNA expression of PSMA was associated with worse overall survival.

**Table 1:** Clinicopathological characteristics of the study cohort (immunohistochemistry cohort)

Parameter	Absolute	Proportion
**Number of patients**	153	**-**
**Number of tumours**	168	**-**
**Age**, years	64.5	-
mean (range)	(20-89)	
**Gender**:		
Female	28	18.3%
Male	125	81.7%
**Cirrhosis**:		
No	71	46.4%
Yes	82	53.6%
Tumor size in mm^*^, mean (range)	51.8 (3-210)	
Histological grade^*^:		
G1	8	4.8%
G2	96	57.2%
G3	59	35.1%
G4	4	2.3%
N/A^1^	1	0.6%
pT-stage^*^:		
pT1	53	31.6%
pT2	43	25.6%
pT3	43	25.6%
pT4	6	3.5%
missing	23	13.7%
pN-category^*^:		
pN0, pNx	159	94.6%
pN1	9	5.4%
Vascular invasion^*^:		
V0	60	35.7%
V1	55	32.7%
Vx	53	31.6%
M-status^*^:		
M0	66	39.3%
M1	26	15.5%
Mx	76	45.2%
**Etiology^2^:**		
Hepatitis B	20	13.1%
Hepatitis C	27	17.7%
Alcohol	40	26.1%
Other	4	2.6%
Negative	34	22.2%
**Follow-up available (overall survival):**		
No	46	30.1%
Yes	107	69.9%
**Mean follow-up time (range), months**	31.0 (1-212)	
**Status at the end of the follow-up (n=107):**		
Deceased	26	24.3%
Alive	81	75.7%

Comments: ^1^ - one patient after neoadjuvant chemotherapy. ^2^ – several patients had more than one etiological factor. ^*^ - this data is presented pro tumour (not pro patient case).

**Table 2:** Univariate and multivariate Cox regression analyses of the FOLH1 mRNA expression in the TCGA hepatocellular carcinoma cohort; overall survival as an end-point (n = 359; number of events = 125)

Parameter	Univariate Cox-analysis			Multivariate Cox-analysis		
	HR	95% CI	p-level	HR	95% CI	p-level
***FOLH1 mRNA expression***						
> median	1.0	-	-	1.0	-	-
< median	1.9	1.3-2.7	0.001	2.1	1.3-3.4	0.002
**pT-stage**						
pT1	1.0	-	-	1.0	-	-
pT2	1.5	0.9-2.4	0.104	1.1	0.6-2.0	0.789
pT3	2.6	1.7-4.1	1.5e-05	2.0	1.1-3.6	0.024
pT4	5.3	2.6-10.6	3.2e-06	3.5	1.2-10.4	0.026
**ECOG performance status**						
ECOG 0	1.0	-	-	1.0	-	-
ECOG 1	1.8	1.1-3.1	0.020	1.8	1.1-3.1	0.025
ECOG 2	3.8	2.0-7.3	5.7e-05	3.2	1.5-6.7	0.032
ECOG 3	9.4	4.2-21.2	6.9e-08	5.0	1.8-14.3	0.002
ECOG 4	38.6	8.5-175.9	2.3e-06	19.0	3.6-100.0	0.0005
**Cirrhosis**						
N/A	1.0	-	-	1.0	-	-
Child A	0.4	0.3-0.6	8.2e-07	1.2	0.6-2.4	0.593
Child B	0.7	0.3-1.5	0.409	2.4	0.9-6.0	0.069
Child C	0.9	0.1-6.4	0.901	5.7	0.7-47.6	0.107
**Postoperative ablation**						
no	1.0	-	-	1.0	-	-
yes	0.37	0.2-0.9	0.029	0.2	0.1-0.9	0.034
**Postoperative systemic therapy**						
no	1.0	-	-			
yes	1.4	0.8-2.3	0.238			
**R-status**						
R0	1.0	-	-			
R1	1.6	0.8-3.3	0.198			
**Vascular invasion**						
no	1.0	-	-			
yes	1.3	0.9-2.1	0.211			
**Histological grade**						
G1	1.0	-	-			
G2	1.1	0.6-1.9	0.774			
G3	1.2	0.7-2.1	0.541			
G4	1.3	0.5-4.0	0.595			
**Gender**						
male	1.0	-	-			
female	0.9	0.6-1.3	0.480			

Comments: TCGA - The Cancer Genome Atlas

### Case report of diagnostic ^68^Ga-PSMA-11 PET/CT in a patient with HCC

**Figure 5:**
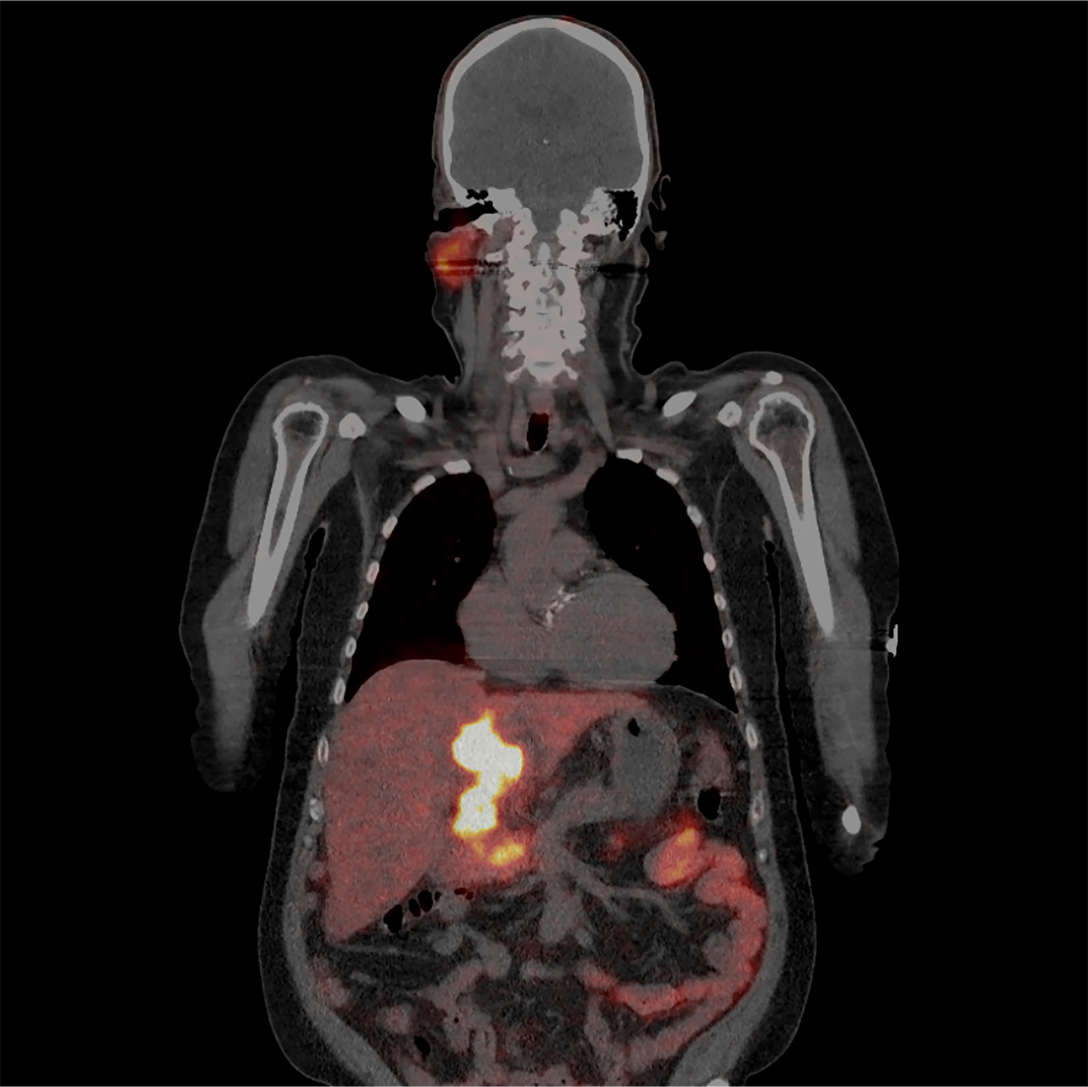
A 62-year-old patient with liver cirrhosis was diagnosed with HCC in 2015. He was primarily treated in December 2015 with radioembolization using ^90^Y-glass microspheres of segment 4 (300 Gy), November 2016 because of a progressive disease the patient received a second radioembolization of the left liver lobe. February 2017 due to further progress of the tumor he started with sorafenib. In August 2017 because of tumor progression a ^68^Ga-PSMA-11 PET/CT was performed to investigate the possibility of a radioligand therapy. Coronal view of the PET/CT scan shows an intense and heterogeneous PSMA uptake in HCCs.

A 62-year-old patient with liver cirrhosis was diagnosed with HCC in 2015. He was primarily treated in December 2015 with radioembolization using ^90^Y-glass microspheres of segment 4 (300 Gy). In November 2016 the patient received a second radioembolisation of the left liver lobe because of a progressive disease. In February 2017 due to further progress of the tumor he started sorafenib. In August 2017 because of tumor progression a ^68^Ga-PSMA-11 PET/CT was performed to investigate the possibility of a radioligand therapy. Coronal view of the PET/CT scan shows an intense and heterogeneous PSMA uptake in HCCs (Figure 5).

## DISCUSSION

This study provides further evidence that prostate-specific membrane antigen (PSMA) is not at all prostate-specific but widely expressed in solid tumors including also hepatocellular carcinoma, which opens up new diagnostic and therapeutic possibilities [1,11]. Although many tumor types express PSMA to certain extent [2–5], the applicability of PSMA as a diagnostic or therapeutic target will probably be limited to those which show a higher than average expression (see analysis of mRNA expression based on TCGA expression data on Figure 1). One of these tumors is hepatocellular carcinoma. Only several case reports to date highlighted PSMA positivity of hepatocellular carcinoma on PET/CT [7–10]. Therefore, we provide here the first comprehensive evaluation of PSMA expression in HCC, benign peritumoral tissue and benign liver tumors.

In our immunohistochemistry study completely negative HCCs accounted only for minority of cases (4.1%), while moderate to high levels of positivity were evident in almost 80% of tumors. Importantly, contrary to many other tumors (renal cell carcinoma, breast cancer, colorectal adenocarcinoma, stomach adenocarcinoma [2–5]) HCCs presented not only with neovascular pattern of expression, but also with a parenchymal, canalicular pattern. This pattern is already seen in benign liver tissue and is also preserved in many malignant lesions. So far, the role of PSMA in benign and malignant tissue is unclear. The demonstration of high PSMA levels in tumor tissue clearly warrants further PET/CT-based studies on the diagnostic performance of PSMA in patients with HCC. Our case of a patient with HCC progressing on multiple lines of treatment confirms the successful applicability of PSMA as a diagnostic *in vivo* agent (Figure 5).

Previously, we described the first case report of radionuclide therapy in a female patient with triple-negative breast cancer [3] and we had expressed concerns that pure vascular expression could significantly reduce the therapy effect due to quicker wash-out of agent and thus absence of a direct effect on tumor cells. Given the above mentioned canalicular pattern in HCC tumors the effect of therapy could be directed not only on vessels but also on tumor cells (analogue to prostate cancer). Together with the overall very high levels of PSMA expression in HCC we assume a better PSMA radionuclide therapy response in these patients.

Additionally, this study demonstrates that PSMA expression may be a novel independently prognostic marker for overall survival when analyzed at the mRNA level with lower expression levels associated with worse survival, which warrants further validation. On protein level we were not able to show this effect, which may be due to the small size and the paucity of events in our cohort which we state as a limitation of the survival analysis.

Other limitation of our study is using overall survival as only endpoint in survival analyses. However, in the TCGA cohort we include ECOG performance status in multivariate models, which allows for corrections with regard to other death causes.

There are several other considerations which should be born in mind while considering PSMA as diagnostic and / or therapeutic agent. Several benign processes / conditions (e.g. sarcoidosis, tuberculosis, regeneration and repair, benign tumors) and a number of malignant tumors are known to be associated with PSMA expression [6,12–15]. Specificity would be probably one of the main concerns and areas of investigation in the future. A second point is the heterogeneity of PSMA expression. HCCs in our immunohistochemistry studies demonstrated significant levels of heterogeneity. The effects of this are not known and should be evaluated in the context of further diagnostic and therapeutic studies. The third point is the abundancy of different PSMA antibodies targeting different epitopes [16], which could influence the diagnostic / therapeutic reproducibility. The fourth point concerning using PSMA as therapeutic target is its relative high expression in benign tissue. Although evidence available from the literature (patients n = 10) and a case report presented here show a very good contrast in PET/CT between most studied lesions (stronger uptake) and surrounding benign liver tissue (lower uptake), mRNA and protein data show an opposite trend with only 10% of tumor samples showing *FOLH1* mRNA expression higher than average in benign tissue. However, it remains to be shown if mRNA/protein expression directly translates into increased PET/CT uptake levels, which might be modulated by different antibody binding capacity/ability, differences in metabolism, wash-out issues, presence of PSMA splice variants or other unknown influences. All this remains unclear and warrants further studies to the function of PSMA in normal tissue and correlation of nuclear medicine and tissue analysis results.

Hepatocellular carcinomas show in general high levels of PSMA expression on tumor vessels and on canalicular membrane of tumor cells. At the mRNA expression level PSMA is also independently prognostic for overall survival. Our case of radionuclide PSMA-diagnostic demonstrates feasibility and efficacy of this modality in a patient with HCC, even after several lines of therapy. One recently published case series (n=7) with majority of patients being therapy-naïve shows similar results with multiple liver lesions, several of them also with PSMA positive metastatic lesions, corroborated by several other published case reports [7,9,10,17]. At that, benign liver conditions (regenerative nodules) in showed only low levels of expression similar to our results which emphasize the potential theranostic role of PSMA in HCC. Kessler et al. also report on the immunohistochemistry results in three patients with tracer uptake in HCC lesions on PSMA-PET/CT [8]. Interestingly, only vascular staining pattern was seen in these three cases. Our study clearly shows that more than 40% of the HCC also express PSMA on canalicular membrane similar to benign liver tissue. At that, an overall expression of PSMA in tumor tissue (RNA analysis) is higher for canalicular pattern compared to pure vascular pattern of expression, even in case of comparable protein expression levels (immunohistochemistry staining). This, as already mentioned above, could have therapeutic implications in contrast to pure neovascular expression and potentially serve as predictive tool on biopsy material. Further prospective studies investigating the diagnostic and therapeutic capabilities of PSMA in HCC are clearly warranted.

## MATERIALS AND METHODS

### Immunohistochemistry cohort

The patient cohort consisted of 153 patient cases with hepatocellular carcinoma treated or evaluated in course of reference pathology examination in the University Hospital of Bonn (1998-2013) with some of them operated on more than once. Clinicopathological characteristics and availability of the follow-up information is outlined in Table 1.

PSMA protein expression was also analyzed in 13 focal nodular hyperplasia (FNH) cases and in 11 inflammatory hepatic adenomas (IHA) from the University Hospital Bonn.

### Tissue microarray (TMA) construction

TMAs were constructed using four tumor cores and two benign tissue cores per tumor, each 1 mm in diameter, arranged in 39 paraffin blocks.

### The Cancer Genome Atlas (TCGA) cohort

Clinical data and normalized mRNA expression (Illumina HiSeq 2000 RNA sequencing platform, Version 2) were extracted for TCGA hepatocellular carcinoma cohort. After rigorous quality control of the database 359 patients (Supplementary Table 1) were available for analysis containing complete follow-up data (overall survival; mean follow-up time 26 months, range 1-123 months). Fifty-two benign liver tissue samples were available. We used vascular normalization of *FOLH1* mRNA expression through mRNA expression of vascular marker (CD34) as described earlier [2]. CD34 was shown to stain virtually all vascular structures within HCC tissue [18]. Tumoral mRNA expression data were also extracted for available tumor types from TCGA to generate Figure 1.

### Immunohistochemistry protocol

TMAs were cut (3 μm) and mounted on superfrost slides (Menzel Gläser, Brunswick, Germany) with subsequent deparaffinization with xylene, gradual rehydration and antigen retrieval by pressure cooking in 0.01 mol/L citrate buffer for 5 min. Slides were incubated with primary PSMA antibody (mouse monoclonal antibody, Dako/Agilent, Clone 3E6; dilution 1:250), counterstained with hematoxylin, and aqueously mounted.

### Immunohistochemistry evaluation

The evaluation of immunohistochemical staining was carried out by two reviewers (YT, AK), who were blinded for clinical data. Agreement was reached in all cases. The staining intensity was evaluated: 1) on the endothelia of tumor vessels (neovascular pattern) using a 4-tiered scoring system (0: negative; 1: weakly positive; 2: moderately positive; 3: strongly positive) for each tumor core separately complemented by percentage of positive vessels; 2) on the membrane of tumor cells (canalicular pattern), using the same 4-tiered system and percentage of positive cells. Agreement was achieved in all discrepant cases between the two reviewers.

### mRNA expression analysis

Ten tumors from 9 patients with HCC from immunohistochemistry cohort as well non-matched 5 benign liver samples were analyzed with regard to mRNA expression of full-length *FOLH1*. Representative 3 μm full sections from blocks with formalin-fixed paraffin-embedded material were submitted to PSMA immunohistochemistry which was evaluated as stated above. One to three 10 μm corresponding sections were used for total mRNA extraction using PureLink™ FFPE RNA Isolation Kit (ThermoFisher Scientific, Waltham, MA, USA) according to manufacturer instructions. mRNA quality control and quantification were performed using a NanoDrop 2000 spectrophotometer (Thermo Scientific, USA). Expression levels of FOLH1 were determined using nCounter platform (NanoString Technologies, Inc; Seattle, WA, USA) and absolute quantification of mRNA expression in counts via nCounter barcoding technology. All samples were titrated to 100 ng of total mRNA amount. Internal negative and positive controls were used for quality control. Negative controls were used for background subtraction (geometric mean) of received expression values. Internal positive controls and four house-keeping genes (*HPRT1, ALAS1, ARF1, PGK1*) were used for normalization of expression values. The sequences of all probes are outlined in Supplementary Table 2.

### Case report of diagnostic ^68^Ga-PSMA-11 PET/CT in a patient with HCC

^68^Ga-PSMA-11 PET/CT was performed in a 62-year-old patient with HCC progressing on multiple lines of treatment from University Medical Center Utrecht, Netherlands

### Ethical considerations

The studies on archive material were approved by the ethical committee of the University Hospital Bonn (No. 167/16). The necessity to provide the informed consent was waived by the ethical committee.

### Statistics

Statistical analyses were performed in R (R Foundation for Statistical Computing; version 3.4.4). Follow-up for TCGA cohort in survival analyses was truncated at 80 months postoperatively.

## SUPPLEMENTARY MATERIALS TABLES



## Abbreviations

FOLH1: folate hydrolase 1
FNH: focal nodular hyperplasia
HCC: hepatocellular carcinoma
IHA: inflammatory hepatic adenoma
PET / CT: positron-emission tomography / computed tomography
PSMA: prostate-specific membrane antigen
TCGA: The Cancer Genome Atlas
