# Supplementary material for: Prostate-specific membrane antigen expression in hepatocellular carcinoma: potential use for prognosis and diagnostic imaging
Source: Oncotarget. 2019 Jun 25;10(41):4149–60. doi: 10.18632/oncotarget.27024 (PMC6609242; doi:10.18632/oncotarget.27024)
Supplement: Supplementary file 1 [file oncotarget-10-4149-s001.pdf]

Prostate-specific membrane antigen expression in hepatocellular carcinoma: potential use for prognosis and diagnostic imaging

SUPPLEMENTARY MATERIALS

Supplementary Table 1: Clinicopathological characteristics of the TCGA hepatocellular carcinoma cohort (mRNA expression)

Parameter	Absolute	Proportion
**Number of patients**	359	**-**
**Age**, years	59.6	-
mean (range)	(16-90)	
**Gender**:		
Female	117	32.6%
Male	242	67.4%
**Cirrhosis**:		
N/A	126	35.1%
Child A	211	58.8%
Child B	21	5.8%
Child C	1	0.3%
**Histological grade:**		
G1	53	14.8%
G2	171	47.6%
G3	119	33.1%
G4	11	3.1%
missing	5	1.4%
**pT-stage**:		
pT1	175	48.7%
pT2	89	24.8%
pT3	79	22.0%
pT4	13	3.6%
missing	3	8.3%
**Vascular invasion:**		
V0	199	55.4%
V1	104	29.0%
Vx	56	15.6%
**Follow-up available (overall survival):**		
No	0	0%
Yes	359	100%

Parameter	Absolute	Proportion
**Mean follow-up time (range), months**	31.0 (1-212)	
**Status at the end of the follow-up:**		
Deceased	125	34.8%
Alive	234	65.2%
**ECOG performance status**:		
ECOG 0	160	44.6%
ECOG 1	81	22.6%
ECOG 2	26	7.2%
ECOG 3	12	3.3%
ECOG 4	2	0.6%
Missing	78	21.7%
**Postoperative therapy:** *Ablation (any type)*	27	7.5%
Systemic therapy	40	11.1%
Chemotherapy	11	3.0%
Targeted therapy	28	7.8%
Both	1	0.3%

Comments: TCGA – The Cancer Genome Atlas

Supplementary Table 2: Probe sequences used for detection of mRNA during nCounter-based mRNA expression analysis in immunohistochemistry cohort

Target	Accession	Position	Target sequence
FOLH1	NM_001014986.2	332-431	GGGCGCTGGTGCTGGCGGGTGGCTTCTTTCTCCT CG-GCTTCCTCTTCGGGTGGTTTATAAAATCCTCC AATGAA-GCTACTAACATTACTCCAAAGCATAA
HPRT1	NM_000194.1	241-340	TGTGATGAAGGAGATGGGAGGCCATCACATTGTA GC-CCTCTGTGTGCTCAAGGGGGGCTATAAATTC TTTGCT-GACCTGCTGGATTACATCAAAGCACTG
ALAS1	NM_000688.4	396-495	AGAAAGCAGGCAAATCTCTGTTGTTCTATGCCCA AAACT-GCCCCAAGATGATGGAAGTTGGGGCCAA GCCAGCCCC-TCGGGCATTGTCCACTGCAGCAGT
ARF1	NM_001024227.1	1371-1470	CAATTCTGCATGGTCACAGTAGAGATCCCCGCAA CTCG-CTTGTCCTTGGGTCACCCTGCATTCCATAG CCATGTGC-TTGTCCCTGTGCTCCCACGGTTCC
PGK1	NM_000291.2	1031-1130	GCAAGAAGTATGCTGAGGCTGTCAC TCGGGCTAAGCA-GATTGTGTGGAA TGGTCCTGTGGGGGTATTTGAATGG-GAAGCTTTTGCCCGGGGAACCAAAGC

Supplementary Table 3: Univariate and multivariate Cox regression analyses of the normalized vascular FOLH1 mRNA expression in the TCGA hepatocellular carcinoma cohort; overall survival as an end-point (n = 359; number of events = 125)

Parameter	Univariate Cox-analysis			Multivariate Cox-analysis		
	HR	95% CI	*p*-level	HR	95% CI	*p*-level
**FOLH1/ CD34 mRNA***						
> median	1.0	-	-	1.0	-	-
< median	1.7	1.2-2.4	0.005	1.6	1.0-2.6	0.050
**pT-stage**						
pT1	1.0	-	-	1.0	-	-
pT2	1.5	0.9-2.4	0.104	1.2	0.6-2.1	0.611
pT3	2.6	1.7-4.1	1.5e-05	1.9	1.1-3.5	0.030
pT4	5.3	2.6-10.6	3.2e-06	3.7	1.3-11.1	0.018
**ECOG performance status**						
ECOG 0	1.0	-	-	1.0	-	-
ECOG 1	1.8	1.1-3.1	0.020	1.6	0.9-2.8	0.083
ECOG 2	3.8	2.0-7.3	5.7e-05	3.4	1.6-7.1	0.0009
ECOG 3	9.4	4.2-21.2	6.9e-08	5.8	2.0-16.5	0.0009
ECOG 4	38.6	8.5-175.9	2.3e-06	21.0	3.9-111.4	0.0004
**Cirrhosis**						
N/A	1.0	-	-	1.0	-	-
Child A	0.4	0.3-0.6	8.2e-07	1.1	0.5-2.2	0.814
Child B	0.7	0.3-1.5	0.409	1.5	0.6-3.7	0.415
Child C	0.9	0.1-6.4	0.901	5.1	0.6-41.7	0.133
**R-status**						
R0	1.0	-	-			
R1	1.6	0.8-3.3	0.198			
**Vascular invasion**						
no	1.0	-	-			
yes	1.3	0.9-2.1	0.211			
**Histological grade**						
G1	1.0	-	-			
G2	1.1	0.6-1.9	0.774			
G3	1.2	0.7-2.1	0.541			
G4	1.3	0.5-4.0	0.595			

Parameter	Univariate Cox-analysis			Multivariate Cox-analysis		
	HR	95% CI	*p*-level	HR	95% CI	*p*-level
**Gender**						
male	1.0	-	-			
female	0.9	0.6-1.3	0.480			

Comments: TCGA - The Cancer Genome Atlas. * - normalization of FOLH1 expression using mRNA expression of the vascular marker CD34.
